# Horizontal Gene Transfer, Fitness Costs and Mobility Shape the Spread of Antibiotic Resistance Genes into Experimental Populations of *Acinetobacter Baylyi*

**DOI:** 10.1093/molbev/msad028

**Published:** 2023-02-15

**Authors:** Aysha L Sezmis, Laura C Woods, Anton Y Peleg, Michael J McDonald

**Affiliations:** School of Biological Sciences, Monash University, Clayton, Victoria, Australia; School of Biological Sciences, Monash University, Clayton, Victoria, Australia; Department of Infectious Diseases, The Alfred Hospital and Central Clinical School, Monash University, Melbourne, Victoria, Australia; Infection Program, Monash Biomedicine Discovery Institute, Department of Microbiology, Monash University, Clayton, Victoria, Australia; Centre to Impact AMR, Monash University, Clayton, Victoria, Australia; School of Biological Sciences, Monash University, Clayton, Victoria, Australia; Centre to Impact AMR, Monash University, Clayton, Victoria, Australia

## Abstract

Horizontal gene transfer (HGT) is important for microbial evolution, but how evolutionary forces shape the frequencies of horizontally transferred genetic variants in the absence of strong selection remains an open question. In this study, we evolve laboratory populations of *Acinetobacter baylyi* (ADP1) with HGT from two clinically relevant strains of multidrug-resistant *Acinetobacter baumannii* (AB5075 and A9844). We find that DNA can cross the species barrier, even without strong selection, and despite substantial DNA sequence divergence between the two species. Our results confirm previous findings that HGT can drive the spread of antibiotic resistance genes (ARGs) without selection for that antibiotic, but not for all of the resistance genes present in the donor genome. We quantify the costs and benefits of horizontally transferred variants and use whole population sequencing to track the spread of ARGs from HGT donors into antibiotic-sensitive recipients. We find that even though most ARGs are taken up by populations of *A. baylyi*, the long-term fate of an individual gene depends both on its fitness cost and on the type of genetic element that carries the gene. Interestingly, we also found that an integron, but not its host plasmid, is able to spread in *A. baylyi* populations despite its strong deleterious effect. Altogether, our results show how HGT provides an evolutionary advantage to evolving populations by facilitating the spread of non-selected genetic variation including costly ARGs.

## Introduction

Bacterial species proficiency for horizontal gene transfer (HGT)—the uptake and incorporation of exogenous DNA—has long been recognized and exploited in laboratory experiments (Avery et al. 1944). However, the sequencing of microbial genomes over the last 20 years has revealed HGT on a scale much greater than anticipated, and highlighted meaningful differences between how evolution works in prokaryotic and eukaryotic species (Ochman et al. 2000; Smillie et al. 2011; Arnold et al. 2022). First, for many prokaryotic species, although all genetic variation originates from de novo mutation, the proximal source of most genetic variants is HGT (Ochman et al. 2000; Smillie et al. 2011; Arnold et al. 2022). Furthermore, it is now widely understood that an individual microbial genome is not simply comprised of a discrete set of genes shared by every individual of that species. Instead, through HGT, microbial genomes draw from a pangenome, with each genome made up of the same set of conserved “core” genes and a number of accessory genes that vary among strains, ecotypes or even individuals within a population (Brockhurst et al. 2019; Domingo-Sananes and McInerney 2021). Finally, computational (Caro-Quintero et al. 2011; Smillie et al. 2011; Garud et al. 2019; Wyres et al. 2019) and experimental evolution approaches (Chu et al. 2018; Maddamsetti and Lenski 2018; Slomka et al. 2020; Power et al. 2021; Nguyen et al. 2022) have shown that entire genes, or sets of genes, can move between species, conferring complex new traits on the host with a single saltation, thereby rapidly increasing the rate of evolution.

However, most horizontally transferred fragments of DNA will not be beneficial and may have neutral or deleterious effects on fitness (Slomka et al. 2020; Nguyen et al. 2022). It follows that the HGT events that can be detected by comparisons of extant genomes are biased toward the small set of horizontally transferred genetic variants that are favored by natural selection. How does the frequent influx of genetic material with a wide range of fitness effects influence the population genetics of a bacterial population? One model proposed that selection in microbial populations is strong, and would rapidly purge HGT variants that did not confer a selective advantage (Berg and Kurland 2002). However, other theoretical results (Novozhilov et al. 2005; Woods et al. 2020) and data from evolution experiments with HGT (Woods et al. 2020; Nguyen et al. 2022) have shown that the frequent transfer of the same genetic variant can provide sufficient evolutionary force to maintain neutral or even mildly deleterious variants in a population. This effect is similar to the effect of gene-flow or migration on a population's gene frequencies, except that the agent of transfer is not an individual organism, but the mechanisms of HGT such as conjugation (Stevenson et al. 2017; Maddamsetti and Lenski 2018), or natural transformation.

Previous studies of the non-selective spread of horizontally transferred variants have been confined to a few experimental studies and model systems, such as *Pseudomonas fluorescens* (Stevenson et al. 2017), *Escherichia coli* (Maddamsetti and Lenski 2018), and *Helicobacter pylori* (Woods 2020 #1990; Nguyen et al. 2022). In this study, we evolve populations of the bacteria *Acinetobacter baylyi* with frequent HGT from the pathogens *A. baumannii* AB5075 and *A. baumannii* A9844. Both *A. baumannii* strains carry resistance to several antibiotics (methods), and we study the dynamics and transfer of these and other genetic elements in the evolving *A. baylyi* populations. We carry out evolution experiments without antibiotic selection to test whether horizontally transferred DNA, including antibiotic resistance genes (ARGs), can spread to detectable frequencies without the life-or-death selective pressures of antibiotics.

## Results

### Experimental Evolution of *A. baylyi* with HGT from Two *A. baumannii* Strains

**Fig. 1.**
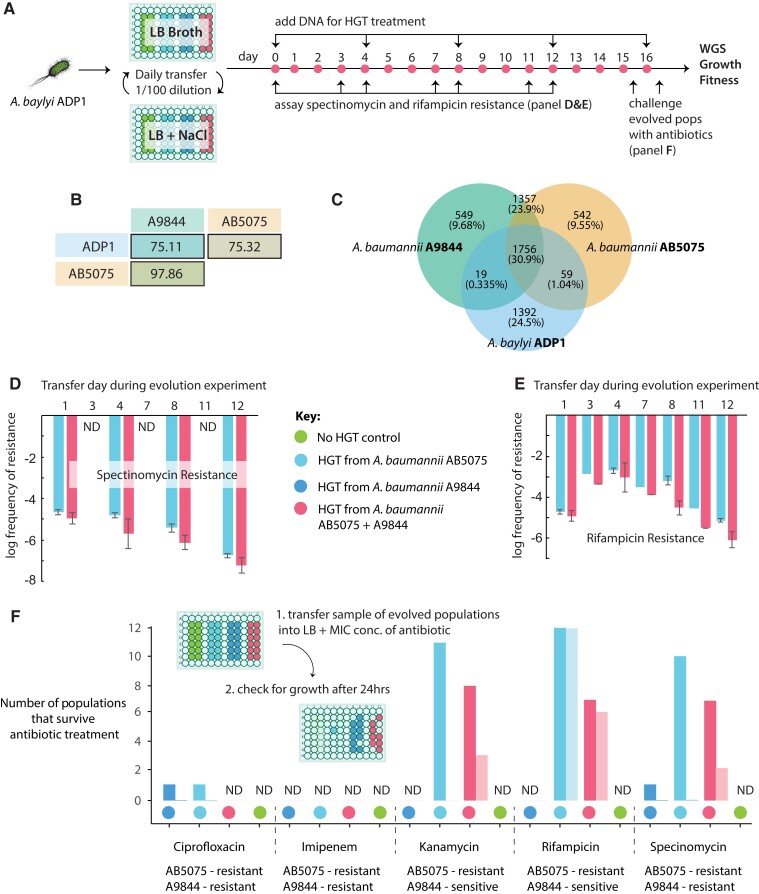
(*A*) Experimental overview. Replicate populations of *A. baylyi* ADP1 were propagated in LB media alternating between 2% and 4% NaCl. HGT treatment populations were supplemented with gDNA from donor strains of *A. baumannii* at the beginning of the experiment and at regular intervals. Accurate well positions are shown on the microwell schematic. (*B*) The average nucleotide identity (ANI) for core genes shared between pairs of genomes used in this study. (*C*) The proportion of shared and unique genes for the donor (A9844 and AB5075) and recipient (ADP1) genomes, where orthologs sharing 95% amino acid identity are considered shared genes. The average frequency of spectinomycin (*D*) and rifampicin (*E*)-resistant colonies across all 12 replicate populations from the AB5075 and mixed HGT treatments. (*F*) The number of evolved populations that could survive challenge with MIC concentrations of ciprofloxacin, imipenem, kanamycin, rifampicin, or spectinomycin antibiotics. Evolved populations were challenged twice: First, at day 15, after a portion of the culture had been taken to inoculate the day 16 cultures (light-colored bars). Second, at day 16, after the HGT treatment (darker colored bars). The light-colored bars show the number of replicates surviving antibiotic treatment 3 days after the day 12 HGT treatment, while the darker colored bars show the number of replicates surviving antibiotic treatment immediately after the day 16 HGT treatment.

We evolved 48 replicate populations of the antibiotic-sensitive *A. baylyi* ADP1, either with or without HGT (fig. 1*A*). The HGT treatment involved adding genomic DNA (gDNA)—and not cells—from two, evolutionarily divergent, multidrug-resistant clinical *A. baumannii* isolates, AB5075 and A9844 (fig. 1*B* and *C*). ADP1 is naturally competent and HGT can be incorporated into an evolution experiment by regularly supplying purified gDNA from a donor strain to the growth media (see Methods). To investigate the effect of the DNA donor, four experimental treatments were included in the experiment: a control in which no DNA was added, HGT from AB5075, HGT from A9844, and a mixed HGT treatment, in which equal amounts of gDNA from both AB5075 and A9844 was added (fig. 1*A*). In each of the treatments with HGT, DNA was added every 4 days. Transformation efficiencies were calculated using samples from half of all replicate populations, on two different antibiotics: spectinomycin and rifampicin. These data confirmed that *A. baumannii* DNA was taken up by *A. baylyi* in the HGT treatment populations, and that independent measures of transformation efficiencies (based on resistance rates to the two different antibiotics) were not significantly different (Spec: 2.32 × 10^−5^± 5.39 × 10^−6 ^cfu/μg, 95% CI; Rif: 1.67 × 10^−5^ ± 5.37 × 10^−6 ^cfu/μg, 95% CI).

### Rifampicin, Kanamycin, and Spectinomycin Resistance Persist in Populations Between HGT Treatments

Previous studies of experimental evolution with HGT in *H. pylori* found that ARGs could establish at low frequencies within a population without direct selection for those alleles (Woods et al. 2020; Nguyen et al. 2022). The persistence of non-selected variants can be due to hitchhiking with selected variants (Nguyen et al. 2022) or the repeated introduction of the same variant by HGT (Woods et al. 2020). During the course of the evolution experiment, we assayed for resistance to rifampicin and spectinomycin by plating a sample taken from the evolving cultures onto antibiotic-supplemented agar plates. Earlier studies have shown that ADP1 can lose competence during an evolution experiment (Bacher et al. 2006; Renda et al. 2015). We regularly measured the resistance frequency which suggested that the evolving populations maintained competence for the duration of this evolution experiment (fig. 1*D* and *E*; Methods). We found that some cells in each HGT population had acquired resistance to spectinomycin but that by the third day after the addition of the DNA, there was no detectable spectinomycin resistance in the population (fig. 1*D*). On the other hand, we found that rifampicin resistance persisted at high levels, for the duration of the experiment, in all populations that received HGT from the rifampicin-resistant strain *A. baumannii* AB5075 (fig. 1*E*). In other words, while both spectinomycin resistance and rifampicin resistance was evident in populations the day after HGT treatment, only rifampicin resistance was maintained in the population for 3 days after the last HGT treatment. At the end of the evolution experiment, we tested whether any of our experimental populations, which had been grown in the absence of antibiotics, had evolved resistance by challenging each replicate with kanamycin, spectinomycin, ciprofloxacin, imipenem, and rifampicin (fig. 1*F*). Note that evolved populations were challenged with antibiotics on day 16, three transfer cycles after the day 12 addition of DNA in the HGT-treated populations, and just prior to the final DNA addition (fig. 1*A*). We found that only those populations that had undergone the HGT treatment could survive in antibiotic-supplemented growth media (fig. 1*E*). However, there was only resistance to rifampicin, kanamycin, and spectinomycin. We assayed for survival again, after the day 16 HGT treatment and found that, in addition to rifampicin, kanamycin, and spectinomycin, two populations were able to survive ciprofloxacin treatment. Imipenem resistance was never detected in evolved populations. These results highlight that the outcomes of evolution with HGT and survival of an antibiotic treatment depends on how amenable a gene is to transfer by HGT, and how long the gene can persist in the population without direct selection.

### Horizontal Gene Transfer, Indels, and Low-Frequency Variants Underlie Evolutionary Change

**Fig. 2.**
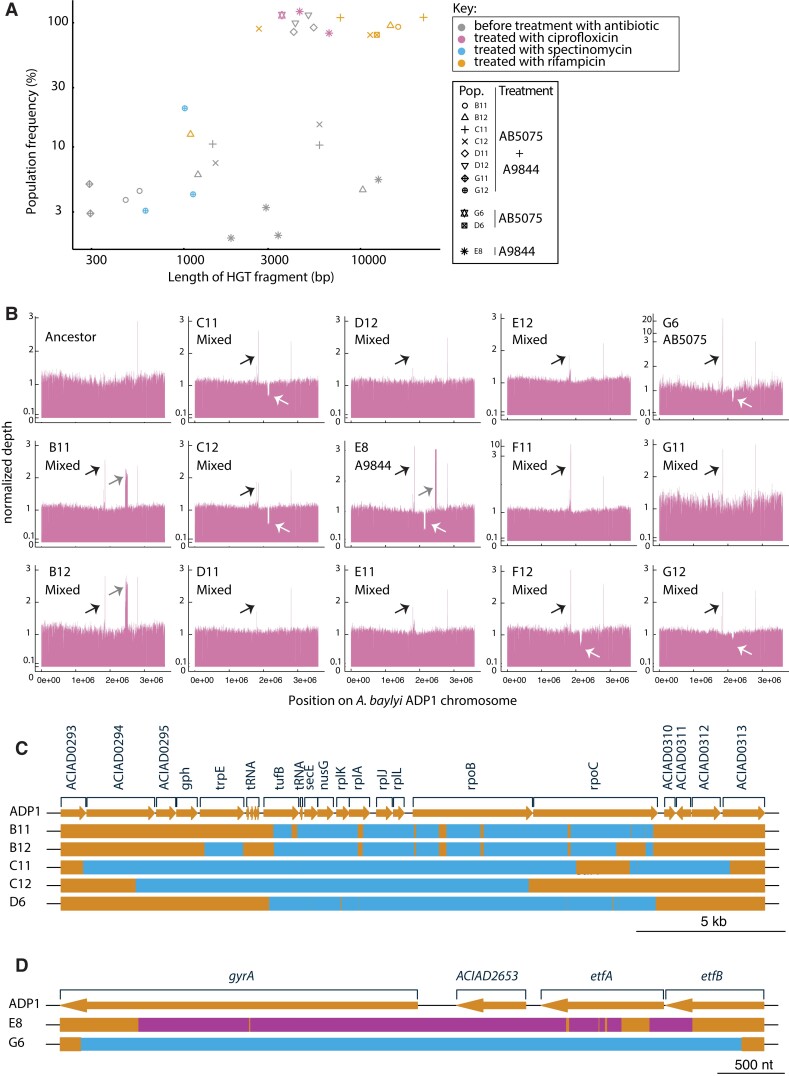
(*A*) The size and population-wide frequency of HGT DNA fragments discovered in evolved populations. The mean frequency of horizontally transferred DNA fragments was greater in populations that had been treated with antibiotic; Wilcoxon Rank Sum, *W* = 198, *P* = 0.004. Note that all 12 sequenced “mixed” HGT treatments were sequenced, and one AB5075 population and one A9844 population were sequenced. (*B*) Coverage plots for 14 sequenced HGT treatment populations. All evolved populations had elevated levels of the activated prophage CRAϕ (black arrow) (Jezequel et al. 2013 #2078; Penda 2016 #2070). Three populations evolved a 30–70 kb duplication (gray arrow) and six populations evolved by deleting a > 40 kb region of the genome carrying another prophage (white arrow). The HGT regions that imparted resistance to (*C*) ciprofloxacin and (*D*) rifampicin. Orange segments represent ADP1 chromosomal DNA, blue segments AB5075, and pink segments A9844 chromosomal DNA. These plots were made using genoPlotR package (Guy et al. 2010). Alphanumeric codes refer to well positions that correspond to each treatment (fig. 1*A*). For example, D6 and G6 correspond to replicate populations from the HGT AB5075 treatment.

To identify the genetic variants that had evolved in HGT and control populations, we carried out whole population sequencing of evolved (no-HGT control and HGT treatment) populations. All 12 “mixed” HGT treatments were sequenced, as were one AB5075 DNA-treated population and one A9844 DNA-treated population. We also sequenced many of the evolved populations that had survived antibiotic treatment. Therefore, multiple samples from each of these evolved populations were sequenced: one sample taken before, and one or more after treatment with antibiotic (fig. 1*A*). In the HGT treatment populations sequenced before treatment with antibiotic, we found that 8 of the 14 populations had a HGT DNA fragment at a population-wide frequency above the threshold of detection (fig. 2*A*, Methods). As expected, each of the populations that had been treated with antibiotic and survived treatment had at least one high-frequency HGT fragment. The mean frequency of horizontally transferred DNA fragments was greater in populations that had been treated with antibiotic (Wilcoxon Rank Sum, *W* = 198, *P* = 0.004; fig. 2*A*). This result supports that most of the genetic variants in our evolving populations segregated at relatively low population-wide frequencies until the introduction of antibiotics drove the spread of the resistance genes.

We also looked for spontaneously arising genetic variants in evolved populations that evolved before the antibiotic treatments. In evolution experiments without strong, life-or-death selective pressures, it typically takes hundreds of generations to fix spontaneously arising SNPs (Good et al. 2017; Leu et al. 2020; Nguyen et al. 2022). In agreement with this, we did not detect any de novo SNPs. However, we did find evidence for the mobilisation of mobile genetic elements, resulting in large genetic duplications and deletions at high frequencies in evolved populations. For example, all evolved populations had elevated levels (2–40-fold) of an activated prophage (CRAϕ) that has been shown to become active in previous evolution experiments with *A. baylyi* ADP1 (Jezequel et al. 2013; Renda et al. 2016). In addition, three populations evolved a 30–70 kb duplication and six populations evolved by deleting a > 40 kb region of the genome carrying another prophage (fig. 2*B*).

### The Cost of Resistance Predicts the Likelihood of Resistance Evolution for Variants That Are Horizontally Transferred into the Recipient Chromosome

We found that rifampicin resistance readily evolved in all of the replicate populations supplied with gDNA from the rifampicin-resistant *A. baumannii* AB5075 (12/12), and in around half of the populations that received the mixed HGT treatment (7/12; fig. 1*C*). In contrast, even though both *A. baumannii* strains AB5075 and A9844 were resistant to ciprofloxacin, resistance to this drug only evolved in 2 out of 36 HGT treatment populations.

The genetic cause of antibiotic resistance in the *A. baumannii* donor strains is well-characterized (Jacobs et al. 2014; Gallagher et al. 2017; Anderson et al. 2018; Gordillo Altamirano et al. 2021), and for some resistant populations, we could readily identify the causes of resistance in the whole population sequence data for antibiotic-selected populations. As expected, 5/5 of the whole-genome sequenced rifampicin-resistant populations had received similar blocks of genetic variants from *A. baumannii* AB5075 *rpoB* (fig. 2*C*) and both ciprofloxacin-resistant populations had received distinct HGT imports of the *gyrA* gene (fig. 2*D*).

**Fig. 3.**
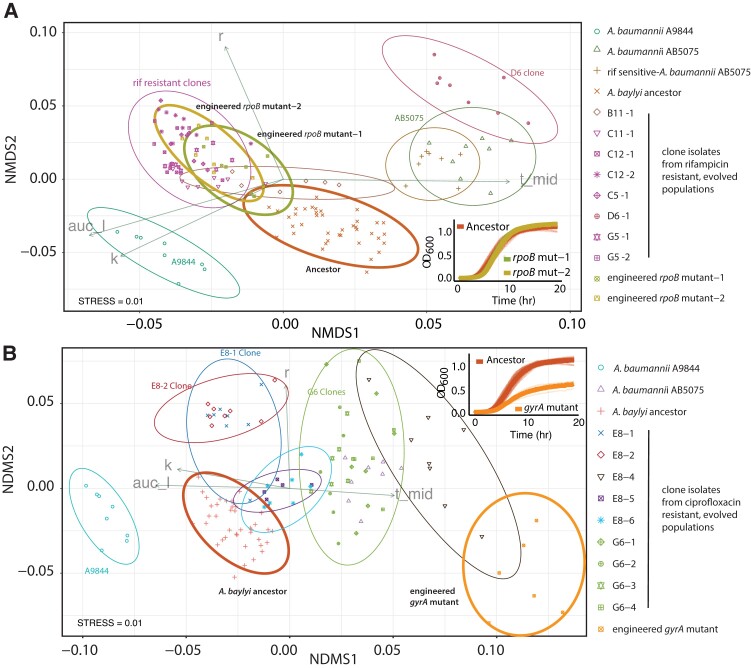
We used Growthcurver (Sprouffske and Wagner 2016) to extract values for R, K, t-mid, and area under the curve (AUC). The growth performance of all strains was compared on two dimensions, using non-metric dimensional scaling. Vectors for R, K, and AUC indicate an increase in growth according to these measures. The increase along the t-mid vector indicates a longer lag time and decreased growth. (*A*) Clones from populations that were able to survive rifampicin treatment were compared with ancestors, donors, and two reconstructed rifampicin-resistant clones. Two reconstructed clones (bold brown and green ellipses) were not different from the ADP1 ancestor (red ellipse). (*B*) The reconstructed ciprofloxacin mutant (orange ellipse) had a longer lag time and reduced carrying capacity compared with the ADP1 ancestor (red ellipse). Each shape depicts an experimental replicate, ellipses show 95% confidence intervals, and ellipses that overlap are not significantly different. Alphanumeric codes refer to well positions that correspond to each treatment (fig. 1*A*). For example, G5-1 and G5-2 correspond to two separate clone isolates from the same population, G5, which is from the HGT AB5075 treatment. Growth curves corresponding to the comparisons shown with bold ellipses are shown inset for panels **A** (bottom right) and **B** (top right).

We engineered fragments of *A. baumannii* AB5075 *rpoB* into the *A. baylyi* ancestor to test whether *rpoB* mutations transferred by HGT cause rifampicin resistance, and any detrimental impacts on growth (fig. 3*A*). We measured cost by carrying out growth assays of ancestor, evolved and engineered strains. Growth curve parameters—carrying capacity (K), growth rate (R), lag-time, and area under the curve (AUC)—were captured using the R package Growth Curver (Sprouffske and Wagner 2016), and the strains were compared using the Non-Metric Multidimensional Scaling R package Vegan (Oksanen 2022). This allowed us to compare all four growth parameters on a single, two-dimensional plot (Methods). This comparison showed that the growth of *A. baylyi* ADP1 strains carrying the *rpoB* mutation was not significantly different than the *A. baylyi* ADP1 ancestor, and therefore that the *rpoB* mutation carries little or no cost.

Since the evolution of ciprofloxacin resistance was very rare (2/36 HGT treatment populations), we hypothesized that the *gyrA* mutation that caused resistance also had a negative impact on growth. We tested this by engineering the *gyrA* mutation into the *A. baylyi* ancestor and performing growth assays. The assays revealed that the *gyrA* mutation is associated with a strong negative fitness effect in *A. baylyi* (fig. 3*B*). Together, these results explain why rifampicin resistance was able to persist in the evolving *A. baylyi* populations without positive selection pressure, and why many of the evolved populations were able to survive treatment with rifampicin, but not ciprofloxacin (fig. 3*A*).

### The Spread of Aminoglycoside Resistance

**Fig. 4.**
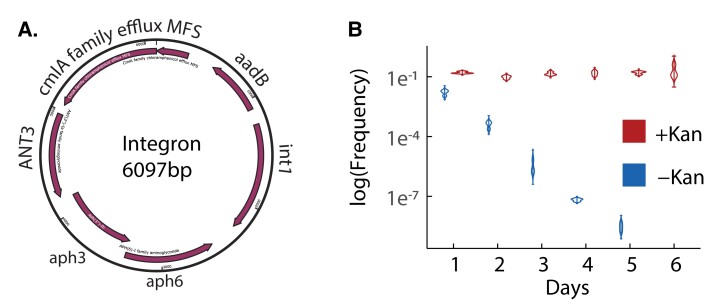
(*A*) The 6020 bp integron is derived from the p1AB5075 plasmid and provides spectinomycin and kanamycin resistance. The *int1* gene encodes an integrase with embedded promoter and *cmlA5* encodes a family chloramphenicol efflux MFS transporter. The remaining genes encode aminoglycoside inactivating proteins, where kanamycin resistance is encoded by *ant(2*″*)-Ia* and spectinomycin resistance is encoded by *aadA3*, *aph(3*″*)-1b* and *aph(6)-Id* based on the ontology from the Comprehensive Antibiotic Resistance Database (CARD) (Alcock et al. 2020). (*B*) Replicate kanamycin-resistant clones were taken from population B12 and passaged for 7 days with or without kanamycin. The rapid loss of kanamycin resistance in growth media without kanamycin suggests that the integron carrying kanamycin resistance confers a strong fitness cost. Notably, even when cultures were supplemented with kanamycin, only around 10% of clones tested were kanamycin-resistant, suggesting that the integron was not faithfully inherited during each cell division.

Many of the evolved populations were able to survive when challenged with the aminoglycoside antibiotics kanamycin and spectinomycin. In *A. baumannii* AB5075, aminoglycoside resistance is encoded on a large (87 kb) plasmid (hereinafter “p1AB5075”). However, genomic assembly of the sequences of the resistant evolved populations did not reveal the expected, full plasmid sequence. Rather, in each case, the only high-frequency horizontally transferred variant identified was a 6020 bp region of the plasmid, containing four ARGs and the *int1* integrase (fig. 4*A*). This region is flanked by att sites recognized by the *int1* integrase. This integron has previously been shown to contribute to tobramycin (an aminoglycoside antibiotic) resistance (Anderson et al. 2018) and virulence (Anderson et al. 2020) in *A. baumannii* AB5075. We looked for evidence of insertion of the integron into the recipient *A. baylyi* chromosome. To do this, we identified reads where part of the read aligned with *A. baumannii* and the other part aligned with *A. baylyi*. Because reads spanning a junction between two non-adjacent genomic regions may be the result of sequencing errors, only those junctions which met depth coverage and region overlap thresholds were considered (Methods). These junctions were not identified in control (non-HGT) lines, providing further evidence that these junctions are not the result of sequencing artifacts. However, while we were able to determine at least one insertion site of the integron for each resistant population, the majority of the integron copies assembled as circular elements with no origin of replication or apparent means of replicating independently of the chromosome (fig. 4*A*). We tested the stability of aminoglycoside resistance by founding replicate cultures with kanamycin-resistant clones taken from an evolved population that had been challenged with kanamycin. We found that kanamycin resistance was completely lost within 6 days of propagation in growth media without kanamycin selection. Interestingly, even for cultures propagated in growth media supplemented with kanamycin, 90% of clones that we assayed were kanamycin sensitive, suggesting that the resistance element was very unstable (fig. 4*B*).

Finally, we found that resistance to imipenem was not transferred in our experimental system. Carbapenem-resistant *A. baylyi* have been isolated from clinical samples, and shown to harbor two beta-lactamase genes (SIM-1 and a OXA-23) (Zhou et al. 2011). This suggests that it is possible for *A. baylyi* to evolve resistance to this antibiotic by HGT. However, it could be that the imipenem resistance genes in the *A. baumannii* donor strains were not readily taken up by *A. baylyi* ADP1 in this evolution experiment, or that multiple loci are required for *A. baylyi* ADP1 resistance to imipenem. A summary of the ARGs from the donor and recipient strains, and the ARGs that were transferred into the evolving populations is shown in Supplementary file 1, Supplementary Material online.

## Discussion

### Cross-Species ARGs Can Persist in Populations Without Selection

Our aim was to carry out an evolution experiment with HGT between species to determine the evolutionary forces that shape the spread of horizontally transferred genetic variation. In previous work, we have shown that neutral or mildly deleterious genetic variants can spread from one *H. pylori* strain to another (Woods et al. 2020). We proposed a model where the repeated introduction of the same genetic variants into a population by HGT can overcome selective constraints that would otherwise suppress the spread of non-beneficial alleles. In this study, we built upon these earlier results by evolving populations of *A. baylyi* with frequent HGT from two multidrug-resistant strains of another species, *A. baumannii*. Our initial aim was to take advantage of the many selectable, ARGs naturally present in these two strains to test whether genetic variants could spread without direct selection. We tracked the spread of genetic variation with two approaches. First, whole population sequencing showed that after 16 days of evolution, 8 out of the 14 HGT-treated populations that we sequenced had acquired genetic variants at frequencies ranging from 1% to 100%. Our second assay was to challenge populations with one of five different antibiotics on day 15, 3 days after the HGT treatment at concentrations above the minimum inhibitory concentration (MIC) for the ancestral *A. baylyi* ADP1. Together, these results support the conclusion that populations with HGT can take up genetic variation from their surroundings and that this genetic variation can improve their capacity to adapt to future changes in the environment. Even though we did not apply antibiotic selection during the evolution experiment, the batch culture conditions and daily dilutions, which corresponded to ∼6.6 generations per day, provide stringent selective conditions compared with natural environments. It is possible that genetic variation is more likely to persist in natural populations, as generation times may be longer due to there being fewer resources and cells are less likely to be randomly removed by the daily dilution process. The capacity for genetic variants to persist in a population for 10–30 generations without selection could correspond to periods of chronological time during which an environmental change is quite likely (Gibson et al. 2018).

Why were HGT populations better able to survive rifampicin than spectinomycin, kanamycin, and ciprofloxacin? In the case of two antibiotics, rifampicin and ciprofloxacin, resistance was attributed to genetic changes at a single chromosomal locus. However, the strong negative growth impact associated with the *gyrA* mutation that causes ciprofloxacin resistance explains its low prevalence—purifying selection rapidly purges this mutation from the population. Meanwhile, the relatively cost-free *rpoB* mutant that causes rifampicin resistance evolved in a relatively large proportion of the populations. These results align with expectations for alleles segregating in a population that undergo similar rates of HGT from donor into host, but that experience different selective forces.

### Mobile Genetic Elements Overcome Fitness Costs to Persist Without Selection

In contrast to *rpoB* and *gyrA*, the genetic element that carries spectinomycin and kanamycin resistance was more difficult to predict. Spectinomycin and kanamycin resistance are conferred by multiple genes that sit between two integrase sites on the *A. baumannii* AB5075 plasmid (fig. 4*A*). We only observed spectinomycin resistance in one population that received HGT from *A. baumannii* A9844, so we focused our attention on populations that received HGT from *A. baumannii* AB5075. Previous studies of this integron locus in *A. baumannii* AB5075 have shown how stress can drive the accumulation of exochromosomal circularized integrons that drive high resistance to aminoglycoside antibiotics (Anderson et al. 2018). Interestingly, while the p1AB5075 plasmid was never detected in any of our experimental lines, the integron was detected at 40–50-fold coverage in all replicate lines treated with kanamycin and spectinomycin. Another study has demonstrated “integrase toxicity” by inserting two Class 1 integrons (from *A. baumannii*) into a neutral chromosomal site in *A. baylyi* ADP1 (Harms et al. 2013). Our observations suggest that the while the plasmid was not able to be transferred into *A. baylyi* in our experiments, the integron and its features—instability, high-cost, and resistance genes—were all successfully transported into *A. baylyi* ADP1 (fig. 4). The genes on the integron (cmlA5, ant(2″)-la, aadA3, aph(3″)-1b and aph(6)-1d) are all not only common across *A. baumannii* genomes, but also each of these genes is found in other pathogenic species (Hernandez-Gonzalez et al. 2022), supporting that interspecies HGT can overcome fitness barriers.

Altogether, these results show how the integron provides a genetic mechanism for a rapid evolutionary response to antibiotics and can be passed intact across species, despite high fitness costs to the host. The measured cost of carrying the integron, that carries aminoglycoside resistance, was much higher than the cost of the *gyrA* mutation that conferred ciprofloxacin resistance. However, unlike ciprofloxacin resistance, kanamycin resistance was able to persist in populations for 3 days without the addition of DNA and was present in HGT populations that were sequenced before challenge with an antibiotic. Another study has shown that a costly plasmid can spread through a population by the driving mechanism of plasmid conjugation (Stevenson et al. 2017). However, in our experimental system, we did not see higher rates of HGT for the plasmid-borne genes (spectinomycin resistance, fig. 1*C*) compared with the chromosomal genes (rifampicin resistance, fig. 1*D*). If rates of transformation are similar, this suggests that the integron may be able to persist in the population, despite its cost, by way of selfish replication mechanisms once it has entered the genome.

## Conclusion

Overall, we have shown that antibiotic resistance can spread between species without selection, and that the evolutionary persistence of resistance depends strongly on costs and the genetic element that bears resistance. In particular, the evolutionary fates of single-copy chromosomally encoded resistance loci are strongly dependent on their fitness effect, while selfish genetic elements are likely to persist despite their cost.

## Methods

### Bacterial Strains and Antibiotic Resistance Profile

The ancestral recipient strain for experimental evolution was isogenic with *A. baylyi* ADP1 wild-type with the following resistance profile, given as MICs: kanamycin 10 µg/ml, spectinomycin 50 µg/ml, imipenem 0.4 µg/ml, ciprofloxacin 1 µg/ml, and rifampicin sensitive MIC 1 µg/ml. Since reported MIC values can depend on the method of measurement and growth conditions, we determined MIC values for the conditions of our evolution experiment. We set up 96-well microplates with LB supplemented with a gradient of antibiotic concentrations (Kan—1, 10, 50; Spec—1, 5, 10, 50, 200; Imi and Cip—0.05, 0.1, 0.2, 0.4, 1; Rif—0.1, 0.3, 0.6, 1, 5). We inoculated wells with a 100-fold dilution of the culture from its carrying capacity in LB media without antibiotics, and monitored growth for 3 days. The well with the lowest concentration of antibiotic that did not show any visually discernible growth was determined to be the MIC for the purposes of the evolution experiment. The two strains that provided donor gDNA for the evolution experiment are *A. baumannii* AB5075 and *A. baumannii* A9844. These strains are clinical isolates shown in previous studies to be resistant to a wide range of antibiotics (Jacobs et al. 2014; Gordillo Altamirano et al. 2021). We verified that each *A. baumannii* strain could grow in media supplemented with antibiotic concentrations that exceed the MIC for *A. baylyi* ADP1. Using this threshold, *A. baumannii* AB5075 was found to be resistant to kanamycin, spectinomycin, imipenem, ciprofloxacin, and rifampicin, while *A. baumannii* A9844 was found to be resistant to spectinomycin, ciprofloxacin, and imipenem.

### Growth Conditions and Evolution Experiment

*A. baylyi* ADP1 was routinely grown on LB agar-based medium at 37 °C. Starter colonies for the evolution experiment were picked from an LB agar plate streaked with *A. baylyi* ADP1. Colonies were inoculated into 132 μl of LB broth in a 96-well microplate and incubated with shaking (500 rpm) for 24 h. Previous studies have found that *A. baylyi* ADP1 loses competence during the period of an evolution experiment (Bacher et al. 2006; Renda et al. 2015). To maintain high and consistent competence throughout the experiment, following incubation, cultures were diluted 100-fold into LB growth media supplemented with 4% NaCl, twice the typical amount. Since our experiment ran for less than 100 generations, we have no evidence that this change slowed the evolutionary loss of competence. HGT treatment populations were propagated by adding 1 μg of gDNA from either 1) *A. baumannii* AB5075 or 2) *A. baumannii* A9844. In the third treatment, a mixture of 0.5 μg of AB5075 and 0.5 μg of AB5075 gDNA was supplied. DNA was added once every 4 days, a time period estimated to provide sufficient time for selection to act on variants before more DNA was added. Control populations did not have any DNA added. Each treatment group consisted of 12 populations, giving a total of 48 evolving populations.

Growth was assayed by growing replicate cultures of the focal strain or population in a microwell plate in LB media. Growth parameters were extracted using R package Growthcurver (Sprouffske and Wagner 2016). Non-Metric Multidimensional Scaling was carried out using R package Vegan (Jari Oksanen 2022) using metaMDS function.

### DNA Sequencing and Assembly

A total of 32 evolved populations were selected for sequencing. Of these, 18 were sequenced prior to antibiotic challenge: 12 from the mixed HGT treatment, 4 from the no-HGT control populations, and 1 population from the AB5075 treatment and 1 population from the A9844 treatment. Another 14 evolved populations were sequenced following antibiotic challenge: mixed HGT populations that survived inoculation with one of rifampicin (4), kanamycin (4), or spectinomycin (4), and one each from the AB5075 and A9844 populations that survived inoculation with ciprofloxacin. We also sequenced one rifampicin-resistant clone isolated from an evolved population that received HGT from AB5075. We prepared whole-genome DNA from samples using the GenElute™ Bacterial Genomic DNA kit (Sigma-Aldrich). Samples were sent to Azenta (Suzhou, China) for sequencing on the Illumina MiSeq next-generation platform. Azenta returned 2 GB of read data, with an average Phred quality score (Q score) of 30 for greater than or equal to 80% of the Illumina paired-end short (150 bp) reads. Adaptor trimming was performed at Azenta, but reads were further filtered and trimmed based on quality score using the BBDuk package (http://jgi.doe.gov/data-and-tools/bbtools/). Since there was not a complete genome for *A. baumannii* A9844, DNA was also prepared for long-read sequencing with the MinION long-read sequencing platform by Oxford Nanopore Technology. Long reads were demultiplexed and base-called according to Wick et al. (2018). The Unicycler (Wick et al. 2017) and Bandage (Wick et al. 2015) software packages were used for hybrid assembly and visualization of short- and long-reads.

The ancestral population was derived from *A. baylyi* ADP1 which has a complete reference sequence available from the NCBI Genome Database so resequencing was sufficient to produce a complete genome sequence (chromosome: NC_005966) (Barbe et al. 2004). Though both donors and the ancestor were each derived from a single isolated cell, the resequencing and variant calling software package *breseq* was used in polymorphic mode with an absolute read count threshold of 2 for all short-read samples to detect any low-level variants that might have arisen during cell culture (Deatherage and Barrick 2014). Each of the *A. baylyi* ADP1, *A. baumannii* AB5075, and *A. baumannii* A9844 genome sequences were annotated with Prokka (Seemann 2014). The genetic content of the strains was compared using the roary pipeline for pangenome analysis (Page et al. 2015) with default settings, in which orthologs sharing at least 95% blastp identity are considered shared genes. Comparisons of the average nucleotide identity (ANI) of the genomes used in this study were carried out using OrthoANIu (Yoon et al. 2017).

Coverage plots were made by first mapping all reads on the recipient genome using BWA-MEM package (Li 2014). Next, SAMtools view were used to convert resulting SAM file to BAM file and SAMtools depth was used to calculate the depth at each base pair in this bam file (Li et al. 2009; Li 2011). Then, the depth at each nucleotide was normalized by the average depth for the whole genome. Finally, 500 bp windows were generated for plotting using the R package ggplot2.

### Discovery of HGT Variants

First, we performed de novo assembly using metaSPAdes (Nurk et al. 2017) to accommodate assemblies at the whole population sequencing level (Bankevich et al. 2012). Next, we removed any contig with average coverage less than five. We then ran a local blastn (Camacho et al. 2009) search to align the filtered contigs to the donor and recipient genome sequences. Manually, we have checked the recombination sites on the recipient genome identified by blastn analyses.

De novo assemblies of some populations revealed a circularized extrachromosomal element which did not encode an origin of replication. Mis-assembly can result in incorrect circularization (Hunt et al. 2015), though inspection of unassembled reads also indicated circularization. A pairwise blastn (Camacho et al. 2009) alignment was performed to confirm that the sequence corresponded to a 6020 bp region of a much larger (∼87 kb) plasmid carried by AB5075. Using *breseq* v 0.35.0 (Deatherage and Barrick 2014), short reads were aligned to a concatenation of the host and recipient genomes, and the junction evidence provided was used to identity cases of recombination between the extrachromosomal element and the host genome using the stringent, default cutoffs to ensure junctions are not sequencing artifacts. Samtools v 1.12 (Li et al. 2009; Li 2011) was used to assess coverage consistency across the region associated with the extrachromosomal element. ARGs were verified and annotated using the ontology from the Comprehensive Antibiotic Resistance Database (CARD) (Alcock et al. 2020).

### Construction of Antibiotic-Resistant Mutants

To engineer ciprofloxacin and rifampicin-resistant mutants, we designed PCR primers to amplify the *gyrA* and *rpoB* genes from each of the donors. Primers for *rpoB* mutant-1 were rpoB(AB5075)-F1: AATGGATGCACCGTACTTATTA and rpoB(AB5075)-R1: CCAATGTTTGGACCTTCAG. The *rpoB* mutant-2 primers were rpoB(AB5075)-F2: ACTTGTCTCCACAAGATTTG and rpoB(AB5075)-R2: CGTGAGTCTTACGACTTAGTC. Primers to amplify *gyrA* were gyrA(AB5075)-F: GATTCCGCACATTACAGAGC and gyrA(AB5075)-R: ACTTCGGTATAACGCATTGC. Next, we grew ancestral *A. baylyi* cells overnight (100-fold dilution) in LB medium supplemented with the PCR amplicons at a final concentration of 4 ng/µl. The next day, 100 µl of the culture was spread on LB plates supplemented with either ciprofloxacin (1 µg/ml) or rifampicin (1 µg/ml) to select for transformants.

### ARG Annotation

We generated predictions for all ARGs in the *A. baumanii* donor genomes, the *A. baylyi* recipient genome, and each of the evolved populations using the CARD database (Alcock et al. 2020). For the recipient and donor genomes, we used the complete assembled genome including plasmids. For the evolved populations, we took advantage of our sequencing depth to included information about antibiotic genes that may have been transferred into the evolving population, but had not fixed (attained a population-wide frequency of 100%). To do this, we carried out a de novo assembly using SPAdes with “–meta” option to accommodate assemblies at the whole population sequencing level for all the evolved populations (Bankevich et al. 2012). Next, we inferred ARGs, using the Resistance Gene Identifier tool from CARD, only considering “Perfect” and “Strict” cases with a ≥ 90 % coverage between the query and the target. For the evolved populations, we only report ARGs that were discovered in the evolving population and were also present in the recipient and/or donor genomes.

## Supplementary Material

msad028_Supplementary_Data

## Data Availability

Assembled genomes and raw DNA sequence files have been deposited at NCBI Bioproject PRJNA865004.
